# Cardiac Metastasis from Prostate Cancer: A Case Study Underlying the Crucial Role of the PSMA PET/CT

**DOI:** 10.3390/diagnostics13172733

**Published:** 2023-08-23

**Authors:** Annalice Gandini, Matteo Bauckneht, Luca Sofia, Laura Tomasello, Giuseppe Fornarini, Elisa Zanardi

**Affiliations:** 1 Medical Oncology 1, IRCCS Ospedale Policlinico San Martino, 16132 Genova, Italy; gandini.annalice@gmail.com (A.G.); giuseppe.fornarini@hsanmartino.it (G.F.); 2 Department of Internal Medicine and Medical Sciences (DiMI), School of Medicine, University of Genova, 16132 Genova, Italy; 3 Department of Health Sciences (DISSAL), University of Genova, 16132 Genova, Italy; matteo.bauckneht@hsanmartino.it (M.B.); luca.sofia97@gmail.com (L.S.); 4 Nuclear Medicine Unit, IRCCS Ospedale Policlinico San Martino, 16132 Genova, Italy; 5 U.O. Clinica di Oncologia Medica, IRCCS Ospedale Policlinico San Martino, 16132 Genova, Italy; laura.tomasello@hsanmartino.it

**Keywords:** prostate cancer, PSMA PET/CT, uncommon metastatic site, intracardiac metastasis

## Abstract

Prostate cancer still represents one of the most frequent cancers and causes of death worldwide, despite the huge therapeutic advances in the last decades. The introduction into clinical practice of prostate-specific membrane antigen positron emission tomography/computed tomography (PSMA PET/CT) has significantly improved diagnostic capacity, allowing for the identification of lesions previously undetectable. The case we are presenting is about a 90-year-old man affected by metastatic prostate cancer and treated with hormonal therapies. At the second progression, the restaging with PSMA PET/CT pointed out a millimetric cardiac intra-atrial metastasis, on which little/scarce literature data are still available. On one hand, this finding confirms the high sensitivity of this technique, which should be preferred over traditional imaging. On the other hand, it suggests that introducing next-generation imaging into clinical practice may provide novel insights about prostate cancer metastatic spread.

A man of 90 years old in good general condition with a past medical history of hypertension, hepatitis A, chronic pancreatitis, and chronic kidney disease (CKD) came to our attention in January 2019, when, due to a prostatic suspicious lesion, he underwent a prostatic biopsy. The histological exam was consistent with a prostatic adenocarcinoma Gleason Score of 5 + 4 = 9/10. A CT scan and a bone scan were performed, and multiple bone lesions were shown, in particular on cervical and dorsal vertebrae and ribs bilaterally. Basal prostate-specific antigen (PSA) was 64 ng/mL. In consideration of the diagnosis of metastatic hormone-sensitive prostate cancer (mHSPC), low volume according to the CHAARTED criteria, the patient started androgen deprivation therapy (ADT) with LHRH-analogue in February 2019, reaching a good biochemical response (PSA = 21 ng/mL). At that time, new hormonal agents (NHAs) were not available in Italy in this setting. The patient was then followed up with a PSA evaluation every 3 months and a CT and a bone scan every 6 months, maintaining a radiologically and biochemically stable disease.

After one year of treatment, we observed biochemical progression, with a PSA = 69 ng/mL; testosterone was suppressed. The reassessment was performed with a CT scan without a contrast agent (for the CKD in the medical history), and a bone scan, showing a progression of the disease with the appearance of new bone metastases. Considering the disease progression and the patient’s generally good clinical condition, on February 2020, he was started on the NHA enzalutamide, while continuing ADT, as the first line in metastatic castration-resistant prostate cancer (mCRPC). This treatment was well tolerated, and the disease remained stable until June 2022, when we observed biochemical (PSA = 328 ng/mL) and radiological progression. At that time, the emerging PSMA PET/CT radiological technique was available in our hospital. Considering that due to the CKD, the patient could not receive a CT contrast agent, it was decided to reassess him with this technique instead of a CT and a bone scan. The patient underwent a [18F]F-PSMA-1007 PET/CT scan with a Siemens Hirez Biograph 16 PET/CT system (Siemens Medical Solutions), a European Association of Nuclear Medicine (EANM) Research Ltd-certified scanner with in-plane FWHM equal to 5.8 mm, a slice thickness equal to 3.75 mm, and an axial field of view equal to 162 mm. A low-dose CT for attenuation correction and anatomical localization was performed using an integrated 16-slice CT scanner. The exam was performed according to the current EANM guidelines [1], showing new bone lesions, particularly at the pelvis, with a pathological fracture in the right acetabular roof due to the presence of pathological newly formed tissue extended to the right iliac wing, and multiple pathological abdominal lymphadenopathies of a maximum diameter of 4 cm.

Therefore, considering our patient unfit to receive chemotherapy due to his general condition, in July 2022, he started a second line for the mCRPC, with abiraterone at the reduced dose of 50%. At the first reassessment, in October 2022, the PSA was 672 ng/mL, and the PSMA PET/CT showed the occurrence of lymph nodal and bone progression on every site. Interestingly, it also showed the appearance of a focal tracer accumulation corresponding to millimetric hypodense tissue located at the interatrial septum (maximum diameter: 10 mm). The maximum standardized uptake value (SUVmax) was 20, as graphically reported in Figure 1, where you can also see the CT, PET, and fused PET/CT corresponding axial images of the cardiac tracer accumulation.

Due to the particular site of tracer accumulation, the images were reviewed in a multidisciplinary meeting. This finding corresponded to a site of minimum tracer accumulation observed in June 2022 (although not significant at that time) and was not present in January 2022. In particular, in Figure 2 are reported the axial CT, PET, and hybrid PET/CT images of, respectively, January, June, and October 2022, with an increasing traced uptake. Although atypical, it was therefore considered compatible with a disease localization; because of the strong suspect and the risk connected to a bioptic procedure, no pathology assessment was performed. Unfortunately, age and severely worsened clinical conditions contraindicated any chemotherapeutic treatment. The patient was referred to best supportive care. He died after almost one year, in May 2023.

Understanding the biology and the natural history of cancers leads to improved treatments and, consequently, survival. In particular, an elevated Gleason Score (9–10) is generally associated with aggressive prostate cancers, and low PSA levels should not exclude highly evolutive disease, since it can be associated with neuroendocrine or undifferentiated histology [2]. The introduction of next-generation imaging has led to significant improvements in the staging and monitoring of this pathology [3]. In early-stage cases, phase III evidence showed that PSMA PET/CT allows for the identification of disease sites that with conventional techniques would be missed due to their small dimensions, thus improving both the staging and restaging of prostate cancer. In these settings, the diagnostic value was validated by histopathology, management changes, and clinical outcomes [4,5,6,7,8].

However, little data are already available regarding the added value of PSMA PET/CT compared to conventional imaging in the advanced disease setting. Indeed, although imaging findings in non-metastatic CRPC (nmCRPC) cohorts propose an added value of PSMA PET/CT in terms of diagnostic accuracy also in the advanced setting [6], a systematic validation has not yet been performed in clinical trials. Current guidelines are based on conventional imaging; staging with new-generation imaging could result in changes in therapeutic strategy. However, the benefits derived from an early detection of progressive disease by PSMA PET/CT and, consequently, the benefits of an early change in treatment, still need to be clarified [9].

The present case report highlights the added value of PSMA PET/CT in the advanced stage of prostate cancer by showing an uncommon cardiac site of tumor spread. Cardiac metastases are exceedingly rare in prostate cancer and may result in increased morbidity and mortality [10].

In a previous case reported by Das et al. [11], an endomyocardial biopsy was performed after the PSMA PET/CT finding of a focal cardiac tracer uptake, and the pathology was consistent with prostate cancer metastasis. With the confirmation by our case of the possibility of myocardial metastasis from prostate cancer, with positive PSMA PET/CT results, the biopsy confirmation, and therefore the invasive investigation, to confirm the progression of disease at this unusual site becomes less necessary and allows for the avoidance of the adverse events associated with this procedure.

Of note, benign PSMA PET/CT cardiac findings have also been previously described in the literature, particularly in the case of lipomatous hypertrophy of the interatrial septum (LHIS) [12]. Although very rare, familiarity with the typical features of LHIS in PET/CT, including the fat-isodense expansion of the interatrial septum, can generally avoid the misinterpretation of metastatic disease. Moreover, in this case, the radiological progression was consistent with the biochemical progression, thus leaning more towards a metastasis from prostate cancer.

## Figures and Tables

**Figure 1:**
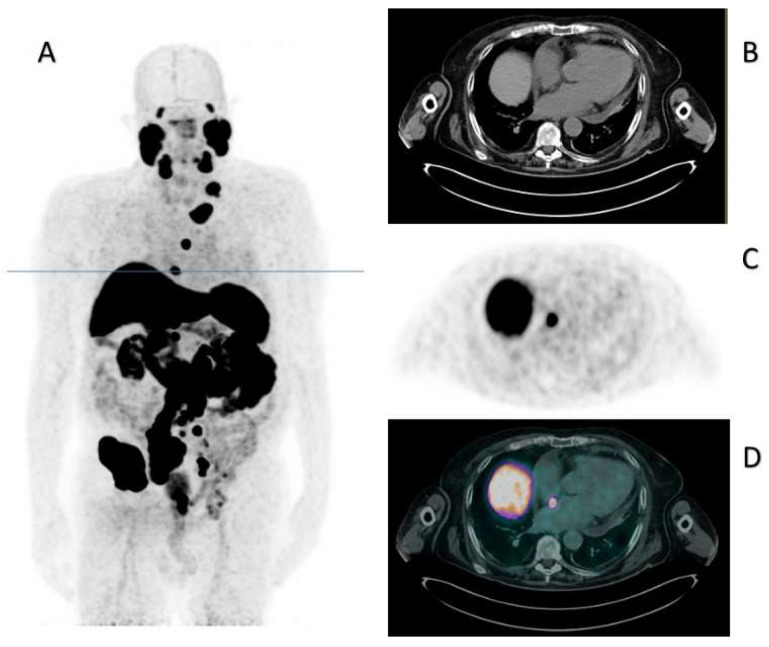
PSMA PET/CT of October 2022. Panel (**A**) displays the maximum intensity projection (MIP), where the blue line indicates the slice corresponding to the cardiac metastasis. Panels (**B**–**D**) indicate the CT, PET, and fused PET/CT corresponding axial images.

**Figure 2:**
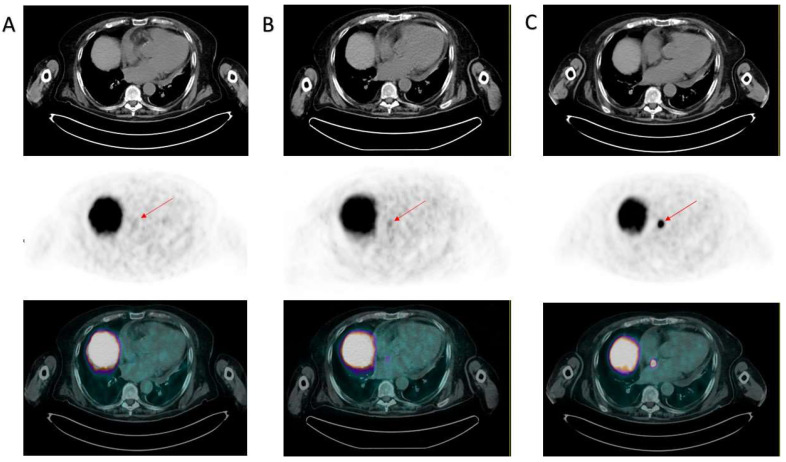
Evolution of the interatrial septum lesion. PSMA PET/CTs from January 2022 (**A)**, June 2022 (**B**), and October 2022 (**C**) are displayed. All panels from the upper to the lower images represent axial CT, PET, and hybrid PET/CT obtained at the same slice displayed in Figure 1. The red arrows indicate the location of the interatrial metastasis, which was absent in January 2022 (**A**), was displayed as a site of minimum (though not significant) tracer uptake in June 2022 (**B**), and was clearly evident in October 2022, with a maximum SUV equal to 20 (**C**).
